# Dissemination of medical research findings among medical researchers in a tertiary institution in Uganda

**DOI:** 10.4314/ahs.v24i3.52

**Published:** 2024-09

**Authors:** Nelson Twinamasiko, Anna Maria Gwokyalya, Joseph Byamugisha, Catherine Misango Precious Namara, David Mpaju, Timothy Mwanje Kintu, Kevin Otim Murungi, Ritah Nantale, Benard Owori, Moses Ocan, Alison Annet Kinengyere

**Affiliations:** 1 Makerere Lung Institute, Department of Medicine, School of Medicine, Makerere University, Kampala, Uganda; 2 Department of Internal Medicine, Uganda Martyrs' Hospital Lubaga, Kampala, Uganda; 3 Aga Khan University Hospital, Nairobi, Kenya; 4 African Center of Excellence in Bioinformatics and Data Intensive Sciences, Infectious Diseases Institute, Kampala, Uganda; 5 St. Francis Hospital Nsambya, Kampala, Uganda; 6 Department of Community and Public Health, Busitema University, Kampala, Uganda; 7 Clinical Epidemiology Unit, School of Medicine, Makerere University College of Health Sciences, Kampala, Uganda; 8 Department of Pharmacology & Therapeutics, Makerere University College of Health Sciences, Kampala, Uganda; 9 Sir Albert Cook Medical Library, Makerere University, Kampala, Uganda

**Keywords:** Dissemination of medical research, Journal Publication, Community engagement

## Abstract

**Background:**

Dissemination of research findings is a key obligation for researchers. It increases access to evidence and the ability to use and apply the evidence. Repackaging of research findings to inform policy and practice is not yet embraced in many low-and-middle income countries that have under-resourced health care systems.

**Objective:**

To determine the methods of communication of research findings by researchers at the Makerere University College of Health Sciences as well as the facilitators and barriers faced while disseminating the findings.

**Methods:**

This was a concurrent nested mixed-methods study among researchers. Key informant interviews and self-administered questionnaires were used. The collected Qualitative data was examined through thematic analysis. Quantitative data were analysed with STATA version 15.0, analysing categorical variables using frequencies and percentages.

**Results:**

Of 176 researchers involved in the quantitative survey, more than half (60%, n=106) were males and 40.9%(n=84/176) were lecturers. The most used dissemination method was journal publications (71.6%, n=126) followed by presentations in conferences/workshops (62.5%, n=110). Twelve researchers participated as key informant interviewees. Themes that emerged included: benefits, facilitators and challenges faced in research communication.

**Conclusions:**

Research findings are commonly disseminated through journals and conference presentations. However, researchers face challenges like financial constraints, time limitations, and misrepresentation of findings.

## Introduction

Dissemination of study findings is one of the inherent responsibilities in the conduct of research^1^. However, few authors move beyond the dissemination of their work in the journal article^2^. Dissemination of study findings increases the reach of evidence, people's motivation, and ability to utilize evidence. Furthermore, when research is designed to improve health, dissemination is critical to the development of evidence-based medicine, and adoption of evidence-supported interventions. When dissemination is lacking, research may be considered a waste of resources; unable to influence positive health outcomes^3^.

Researchers have used traditional outlets to disseminate their research findings: through article publication in peer reviewed journals and book chapters; through dissemination seminars; and through conference presentations. However, these modes often confine audiences to research communities and fellow academicians^2^, yet there are a number of other audiences that would apply the findings to increase the impact of research in practice, and improve the lives of people who use health and social care services.

A study on factors influencing the utilization of research findings by health policy-makers in Mali indicated that limited access to research findings hindered its use^4^. A Nigerian study revealed that policymakers and communities have limited and challenging utilization of research findings^5^. Unless research is adequately reported, the time and resources invested in conducting it are wasted^6^.

Laura et al.,^7^ revealed that medical articles reported in The New England Journal of Medicine and in The New York Times receive about 73 percent more citations in medical reports than articles reported elsewhere. They argued that researchers who can successfully disseminate their findings via media outlets, are more likely to communicate the value of their work to any audience. A study by Steven Keen & Les Todres proposed other ways that go beyond the forms of dissemination that traditionally serve academic communities and attempt to address the communicative concern of research findings 2,7. The study proposes drama, dance, poetry, websites, video and evocative forms of writing.

Repackaging of health research findings to inform policy and the practice of medicine has been slow in many low and middle-income countries^8^. In resource-constrained countries like Uganda, research uptake is affected by limited research-to-user connection^9^. In addition, there is a lack of existing information on the different effective means of dissemination researchers in resource-constrained countries can undertake. Therefore, this study aims to enhance the understanding of research dissemination practices in Uganda, ultimately contributing to the more effective translation of research into practice, policy, and improved health outcomes.

## Methods

### Study design

A concurrent nested mixed-methods study was conducted from April to May 2023 in order to comprehensively understand the different research dissemination methods used by researchers in Uganda and their facilitators and barriers. Data integration and reporting were conducted in-line with JARS-Mixed Methods Article Reporting Standards^10^.

### Study setting

Study was carried out at Makerere University College of Health Sciences (MaKCHS), the oldest Medical School in East Africa, which is located on Mulago hill within the Mulago National referral hospital complex, northeast of Kampala city. The college is comprised of four schools and over 40 units; with a total of 484 staff and a student population of about 2,500 people. Both faculty and students are involved in research at the college making it renowned for research excellence and having a commendable ranking among the top 10 medical schools in Africa.

### Study population

The study targeted researchers whose projects had received ethical approval within the last 5 years from any of the Research Ethical Committees (RECs) at the College of Health Sciences (Infectious Disease Institute REC, School of Public Health REC, School of Medicine REC, School of Health Sciences REC, and School of Biomedical Sciences REC). The lists of these researchers were obtained from the administrators of the different RECs at the college.

### Sample size estimation and sampling

For the quantitative data, we used the Kish-Leslie formula for single proportion^11^ for sample size calculation. We enrolled 176 participants at a desired precision of 0.05 since the proportion of researchers who had disseminated their findings was unknown; P was set at 0.5. Convenience sampling was carried out to obtain the required number of participants, based on the accessibility and willingness to participate in the study.

For the qualitative data, we didn't predetermine the sample size, but used the data saturation principle^12^. Twelve key informants were interviewed until no new information was obtained. Participants sampling was based on their research experience and willingness to participate.

### Data collection

A pretested self-administered questionnaire was used to collect quantitative data whereas a pre-tested key informant interview guide was used to collect qualitative data. The results of the pretest were not included in the analysis.

Four trained research assistants of medical background collected the data. For the interviews participants were contacted for appointments, and thereafter held physically or via Zoom according to the participants' preferences. No third parties were present during the 30–40-minute interviews to ensure privacy. Study objectives were shared and consent was obtained before each interview. Interviews were audio recorded and transcribed clearly, with no need for participant clarification.

### Data analysis

#### Quantitative analysis

Data ware entered in Epi-data software ver 3.1 (EpiData Association, Odense, Denmark) (13). Double data entry and validation were done to ensure correctness and completeness of data. The data was thereafter exported to Microsoft excel 2016 and cleaned, then analyzed in STATA ver 15.0 (StataCorp LLC, College Station, TX, USA). Categorical data were presented as frequencies and percentages.

#### Qualitative analysis

Interview audio recordings were transcribed into Microsoft Word. The principal investigator and two assistants each read the transcripts thrice for familiarity. Thematic analysis was used for data interpretation.

The team created initial codes from three transcripts and developed a codebook. This was applied to the remaining transcripts by various team members for diverse analysis. Discrepancies were resolved through discussions to ensure coding consistency. After coding, the codes were abstracted into sub-themes and themes, linked back to the research questions.

### Ethics statement

Ethical approval was obtained from the School of Medicine REC (approval number: MakSOMREC-2021-235). The study was further reviewed and clearance to conduct it in Uganda was obtained from Uganda National Council of Science and Technology (registration number: HS2881ES). Each participant gave informed consent before data collection. The manuscript doesn't include any data that could reveal the participants' identities.

## Results

### Characteristics of the participants

Majority of the participants were male (60.2%, n=106), aged between 45 to 49 years. Almost half the participants were lecturers (40.9%, n=72) Table 1.

**Table 1:** Characteristics of the participants

Characteristic (n=176)	Frequency, n (%)
**Age (Years)**	
<35	46 (26.1)
35 to 44	49 (27.8)
45 to 59	61 (34.7)
>60	11 (6.25)
Not answered	9 (5.11)
**Gender**	
Female	70 (39.8)
Male	106 (60.2)
**Academic rank**	
Lecturers (Senior and Assistant)	72 (40.9)
Postgraduate students	51 (29.0)
Associate Professor	17 (9.7)
Professor	13 (7.4)
Others*	23 (13.1)
**Affiliation**	
School of Medicine	61 (34.7)
School of Health Sciences	9 (5.1)
School of Biomedical Sciences	58 (32.9)
School of Public Health	27 (15.3)
School of Dentistry	3 (1.7)
Other**	18 (10.2)

* Other academic ranks mentioned included volunteer senior advisor, consultant, researchers, senior consultant, project coordinator, project staff, chief laboratory technician, senior research fellow and research assistant.

** Other affiliations included Makerere Lung Institute, Infectious Diseases Institute.

### Dissemination of medical research findings among researchers at Makerere University

In the last 5 years, 89.8% of the researchers (n=158/176) had completed research projects and 98.1% of these (n=155/158) had disseminated their findings. Additionally, more than half (68%, n=68/100) of the participants with ongoing projects, had disseminated their preliminary findings.

**Figure 1:**
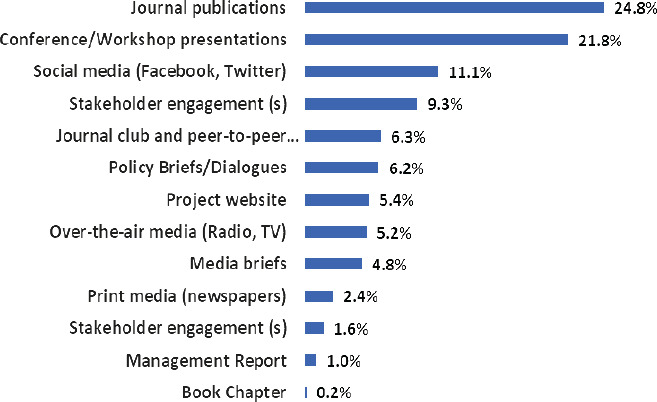
Channels of dissemination used by researchers to disseminate medical research findings at MakCHS

The most used mode of dissemination of research findings was through journal publications (n=125, 24.8%) followed by conference/workshop presentations (n=110, 21.8%) Figure 1.

The target audience for dissemination of these findings included academicians (69.9%, 123/176) and the research community (69.3%, 122/176). Over a third, 39.8% (70/176) of the researchers reported disseminating their findings to the public and over half ((51.7%, n=91/176) disseminated to policy makers. Majority, 98.9% (174/176) of the researchers agreed that dissemination of research findings is important (Table 2).

**Table 2:** Dissemination of research findings among medical researchers at MakCHS

Variable	Frequency (%)
**Have current running research project (s)**	
Yes	144 (81.8)
**Role in the research project/grant**	
Principal investigator	90 (62.5)
Co-principal investigator	26 (18.1)
Research fellow	20 (13.9)
Co-investigator	3 (2.1)
Other***	5 (2.7)
**Have preliminary findings from current research project (n=144)**	
Yes	100 (69.4)
**Disseminated the preliminary findings of the research project (n=100)**	
Yes	68 (68.0)
**Type of journal, if published preliminary findings from current project (n=68)**	
Open access journal	67 (98.5)
Restricted access journal	3 (4.4)
**Location of conference, if presented preliminary findings of current project in conferences/workshops (n=68)**	
International	44 (64.7)
Local	30 (44.1)
**Have research project (s) completed in the last 5 years (n=176)**	
Yes	158 (89.8)
**Disseminated findings from project (s) completed in the last 5 years (n=158)**	
Yes	155 (98.1)
**Type of journal, if published findings from project (s) completed in the last 5 years (n=155)**	
Didn't publish	21 (13.6)
Open access journal	129 (83.2)
Restricted access journal	5 (3.2)
**Target audience for research findings (n=176)**	
Academia	123 (69.9)
Policy/decision makers	91 (51.7)
Health care workers	44 (25.0)
General public	70 (39.8)
Research community	122 (69.3)
Others****	13 (7.4)
**View of research findings dissemination**	
Not important	2 (1.1)
Important	174 (98.9)

*** Other roles included; administrator, and student.

**** Other target audiences were; Ministry of Health, Funders, National TB Program, Undergraduate and Graduate students, District Health Officials, research participants, study stakeholders, and people who contribute to death and birth registration

## Qualitative results

Three themes and 7 sub-themes were identified from the data collected. The themes included; “*Benefits of medical research communication*”, “Facilitators of Medical research communication”, and “*Challenges faced in medical research communication*”.

### Theme 1: Benefits of medical research communication

**This theme explores the merits associated with effective communication of medical research findings.**

Community benefits: Effective communication of medical research supports policy-making, healthcare improvements, and societal understanding. It fosters informed decisions, community interventions, public trust, and stimulates research collaboration and this was evident in their quotes;

*“… you want them to understand the results of your study findings and also probably to influence their health behavior if your results have data that indicates that there should be a change in somebody's self-behavior to take up your research findings.”* (Interviewee 9)

*“It is important to engage the community in research communication. It empowers them to hold leaders accountable, make positive changes for health, and have more ownership of generated interventions”* (Interviewee 2)

**Individual benefits:** Effective research communication boosts career progression, visibility, and credibility. It fosters collaboration, interdisciplinary research, and professional growth. It also enhances funding opportunities and positions researchers as field experts. Examples of their narratives are the following quotes;

*“You also get people asking you about the work you did once you have published. You get people requesting you to go and present your work at conferences. Because most times when you finish research, the research may raise more questions.”* (Interviewee 7)

*“The research conducted by myself and my students has facilitated their completion of academic programs, including masters and PhDs. My research has furthered my academic career, enabling me to write additional projects, apply for grants, and achieve promotion.”* (Interviewee 8)

**Institutional benefits:** Effective communication of medical research at Makerere University has boosted its reputation, rankings, and collaborations, leading to increased visibility, funding, and partnerships, thereby enhancing its institutional standing. The following quotes are examples;

*“The research done at this college and all the other colleges have collectively improved Makerere University ranking globally.”* (Interviewee 2)

*“Funded research has enhanced the college's research management capacity. The college, with its top-tier institutional review boards and trained managers, now offers graduate programs in research administration…”* (Interviewee 3)

### Theme 2: Facilitators of Medical Research Communication

This theme identifies factors facilitating effective medical research communication through both digital and traditional media.

Facilitators for Using Digital Media: Digital media is favored for its affordability, worldwide accessibility, and relevance. It enhances global collaboration and knowledge sharing.

*“…journals efficiently reach a broader, global audience, enhancing the impact of your work beyond your research area.”* (Interviewee 5)

*“It's effective as it keeps me updated on my profession through the latest publications, thus facilitating knowledge exchange.”* (Interviewee 11)

However, some participants think channels such as publications are overrated yet their effectiveness is questionable and used because it is normative in academia;

*“Honestly, I think it is just one of those ways and being in academia it is one of the requirements. So, its effectiveness; I don't know. It is just one of those things that is on your CV. So, in terms of being effective, probably it is not.”* (Interviewee 12)

Facilitators for Using Traditional Media: Researchers opted for traditional media dissemination to engage local communities effectively. Traditional channels provide a familiar platform for facilitating behavioral changes. Prioritizing community engagement ensures stakeholder involvement and understanding of research findings, establishing trust and credibility

*“… but also, the community is bound to respect you if you go back and you want to do a follow-up study because they already know you and you already penetrated the community it is easier. But if you go, do your research and go away and then for whatever reason you need to go back and ask they will be like, “but last time you never even bothered to tell us what you found”*. (Interviewee 7)

*“Because remember your community is your target population and you are using your findings to infer what you have found from this target population to the bigger study population so if you want to change; if your recommendations are to change anything, it starts with that group.”* (Interviewee 1)

### Theme 3: Challenges Faced in Medical Research Communication

This theme addresses the obstacles encountered during communication of medical research findings.

Challenges with Digital Media: Digital media creates challenges for medical researchers, including lengthy peer review processes, high publication costs, and limitations in audience reach. It also perpetuates publication bias and discourages open sharing and innovation due to fear of idea theft and malicious peer review.

*“One journal remained with my paper for so many months and finally told me sorry, we cannot accept it; we failed to get another reviewer; So, publishing in journals can delay; it can be a deterrent; and discourage newcomers, it can be quite stressful for people who are trying to get a knack of it.”* (Interviewee 11)

Challenges with Traditional Media: Traditional media for medical research communication faces challenges like potential public misunderstanding due to technical language, time-consuming stakeholder engagement, limited reach due to accessibility issues and prohibitive costs of options like radio talk-shows, as well as difficulties in community engagement, especially when financial incentives are required and budgets are limited.

*“Then sometimes it is really hard to communicate some technical terms in a manner that people will understand so you find yourself explaining over and over again and people will not grasp the concep*t.” (Interviewer 5)

“Time is a major challenge; you finish one project and then start another; so, at the end of it all you don't have time to package your material and meet the people you intend to disseminate it to.” (Interviewee 11)

## Discussion

Bridging the research-practice gap involves reducing barriers and enhancing research dissemination to stakeholders. Most investigators in this study shared their findings through journals and conferences, targeting academics and researchers, mirroring a similar U.S. study by Heatherlun et al. 14.

From our study, most medical researchers use journals as the means of disseminating their work. Previous studies reveal that while various dissemination methods exist, peer-reviewed journals and professional meetings are particularly popular 14,15. However, these passive approaches to dissemination are often ineffective in changing practice 16-18.

Keen et al^19^, proposed unconventional methods like drama, dance, poetry, websites, videos, and evocative writing to complement traditional methods of research dissemination. Although many researchers from our study didn't fully utilize these alternative methods, they acknowledged their effectiveness.

From this study, researchers agreed that besides academicians, the broader public, policymakers and implementers should be recognized as key beneficiaries of research. Sharing research findings with the community members who would benefit most is expected to promote more equitable knowledge distribution^20^.

Our study found that sharing research findings benefits both individual researchers and institutions. Disseminating research findings enables increased collaboration opportunities and academic promotion, consistent with previous studies 21,22. While these dissemination opportunities do exist, such efforts are not widespread and consistent. Creating a system to reward scholars disseminating their research will greatly increase the vigor and desire for dissemination^23^.

Researchers often consider a project successful if it leads to a peer-reviewed publication. However, our study revealed challenges related to this traditional research communication method. Specifically, a lack of mentorship or training in academic writing was reported to hinder the publication process. Studies have reported that healthcare professionals usually receive little or no formal training in writing^24^,^25^. This deficiency is known to complicate manuscript development, especially for emerging researchers like residents, potentially leading to data remaining in file cabinets and discoveries going unshared 26.

Time-intensive nature of the publishing process was reported to compete with researchers' numerous responsibilities, particularly those juggling multiple projects alongside clinical practice. This aligns with findings from Edwards 26 that concur on the challenge faced by many researchers, in finding sufficient time for dissemination^26^.

Researchers highlighted financial constraints related to organizing conferences, dissemination meetings and information translation. This limitation is similar to that found by Florence Upenyi et al.^27^ in a review that mentioned lack of resources to support translation and dissemination of research findings.

The complexity of disseminating research to communities was also recognized from our study as a deterrent to dissemination of research findings. Similar studies also mentioned challenges in dissemination to the general public arising from the intricate organization of local communities and their isolation from one another 28,29. Additionally, the lack of integration of research with local customs and cultural values poses a common barrier to communication of research findings 29,30. Researchers in our study expressed similar sentiments especially when addressing myths or unhealthy cultural practices that challenge long-standing beliefs.

Policymakers were noted to usually be the most important adopters of research findings and engaging them in dissemination meetings was believed to be one of the easiest ways of raising their awareness of relevant research findings as many lack time to find and read research articles 31,32. However, apart from difficulties in mobilizing policymakers to dissemination meetings, it is equally difficult to capture their interest in research findings particularly when findings do not align with political agendas. This gap between researchers and policymakers is attributed to poor collaboration between researchers and policymakers 32 and treating national-level stakeholders as second audiences 30.

Research findings are often misinterpreted, particularly in media. The responsibility of accurate dissemination was found to typically fall on the researcher, ensuring stakeholders receive correct information.

## Strengths and limitations

This study stands out as one of the initial assessments of research communication by researchers at MakCHS. The inclusion of a qualitative aspect provided an in-depth understanding of the challenges encountered by researchers in communicating their research findings.

While the sample size calculation ensured representativeness, there may still be challenges in achieving a truly representative sample due to selection bias. The study was limited to researchers at Makerere College of Health Sciences, excluding researchers affiliated to other universities hence limiting its generalizability.

## Conclusion and recommendation

Peer-reviewed publications and conference presentations remain the predominant methods for disseminating research findings. Challenges faced by researchers in reaching wider audiences include financial constraints, time limitations, lack of mentorship, inadequate stakeholder engagement, complexity of local communities, misinterpretation and misrepresentation of findings. Researchers are encouraged to incorporate alternative formats of dissemination alongside traditional methods to reach a broader audience and enhance the translation of research into practice.

## Data Availability

Data and supplementary material will not be made available online, but the corresponding author can be contacted to discuss sharing.
